# Electrokinetic remediation of heavy metals from municipal solid waste incineration fly ash pretreated by nitric acid

**DOI:** 10.1098/rsos.180372

**Published:** 2018-08-01

**Authors:** Huilin Li, Faheem Muhammad, Yujie Yan, Manli Zhang, Binquan Jiao, Lin Yu, Dongwei Li

**Affiliations:** 1 State Key Laboratory of Coal Mine Disaster Dynamics and Control, Chongqing University, Chongqing 400044, People's Republic of China; 2 City College of Science and Technology, Chongqing University, Chongqing 400044, People's Republic of China; 3 Chongqing Solid Waste Management Center, Chongqing 401147, People's Republic of China

**Keywords:** electrokinetic remediation, fly ash, heavy metals, pretreatment

## Abstract

Municipal solid waste incineration (MSWI) fly ash has a high concentration of heavy metals (HMs) which are hazardous to the environment. Moreover, it has high pH and buffering capacity which hinders the removal of HMs. Another constraining factor is the considerable fraction of HMs which exist in oxidizable and reducible states. The acid pretreatment of MSWI fly ash is a key solution to this problem. Therefore, the current experiment is carried out to evaluate the effect of acid pretreatment of MSWI fly ash and reaction/proposed time on the removal efficiency of HMs through an electrokinetic experiment. The leaching experiment results show that acid pretreatment has increased the desorption/release of heavy metal ions (Pb^2+^, Cd^2+^, Cu^2+^ and Zn^2+^). It enhances the migration of HM ions in electrolytic cells which get precipitated at the cathode, thereby increasing the removal efficiency of HMs in the electrokinetic experiment. Moreover, it is found that prolonged proposed time (12 d) has significant effect on the removal efficiency of HMs. Finally, it is concluded that acid pretreatment and prolonged proposed time have enhanced the removal electrokinetic remediation of HMs which is carried out via three processes, i.e. desorption (enhanced by acidification), migration and precipitation.

## 1. Introduction

Recently, municipal solid waste (MSW) has increased owing to rapid urbanization throughout the world. MSW is a hazardous material owing to the presence of heavy metals (HMs) such as copper, lead, cadmium and zinc. Therefore, proper treatment of MSW is necessary before its disposal; otherwise, its consequences on the environment are serious [1–3]. Several techniques have been employed for the disposal of solid waste such as composting, landfill and incineration [4,5]. Among them, incineration has been widely used which has reduced the volume of waste besides removing pathogens. Basically, in this technique, solid wastes are burnt at high temperature which results in the volatilization of HMs and are accumulated in their by-products. Therefore, special attention is required for the safe disposal of by-products of incineration which is called municipal solid waste incineration (MSWI) fly ash [6–11].

A number of techniques, such as physical separation and isolation, flushing, washing, toxicity reduction, solidification/immobilization and phytoremediation, are used to treat the HM pollution [12]. However, a number of limitations are associated with these techniques such as partial removal of HMs, high consumption of reagents and energy, low selectivity and generation of secondary wastes. These problems are highlighted because of the high buffering capacity of MSWI fly ash [13].

Keeping the above points in view, electrokinetic remediation technology is widely used to address HM pollution [14]. It can be used for both *in situ* and *ex situ* remediations which have achieved excellent effects in detoxifying contaminated soil, mud and water [15–18]. Thus, it is considered that electrokinetic remediation technology has significant potential for the treatment of MSWI fly ash. Generally, the electrokinetic remediation process is carried out in the electrolytic cell/chamber which consists of a sample area, a voltage source and two electrodes, i.e. cathode and anode. These electrodes are connected with a voltage source and placed in the sample area to generate an electric field which helps in the migration of contaminants/HMs. The remediation mechanism is completed in four steps which are acidification, desorption, migration (electromigration, electro-osmosis and electrophoresis processes) and precipitation of metal ions [19–21].

As mentioned earlier, MSWI fly ash has high buffering capacity which limits the desorption of metal ions. Actually, MSWI fly ash has numerous oxides, such as SO_3_, SiO_2_, CaO_2_ and Al_2_O_3_, and several kinds of metallic oxide which react with water and generate carbonate and hydroxide compounds [22]. These compounds are responsible for the high pH of MSWI fly ash. Consequently, the desirable effect of electrokinetic remediation on MSWI fly ash cannot be achieved owing to the limitation of the desorption process [23]. Traina *et al*. [24] treated the MSWI fly ash by electrokinetic remediation and they found that pH increased during the electrokinetic remediation which limits the removal rate of HMs. In another study, Liao *et al.* [25] prewashed the MSWI fly ash with water to check the removal efficiency of Pb, Cd, Cr and Cu. They have not achieved the desirable results and failed to remove the HMs existing in oxidizable and reducible states. However, Pedersen *et al*. [26] have pretreated the MSWI fly ash with ammonium citrate to remove the Cu, Zn, Pb, Cd and Cr from MSWI fly ash via electrodialytic remediation. They have achieved good results after 69 days and found that it is also not an efficient way to remove HMs. Thus, it is necessary to control the pH of the fly ash before the electrokinetic remediation process which will enhance the removal efficiency of HMs by increasing the desorption and migration of ions.

pH can be controlled by the addition of acid in the fly ash sample before being subjected to electrokinetic remediation [27]. During electrokinetic remediation, pH is controlled through an ion exchange membrane which is placed between the electrolytic solution of the cathode region and sample area. This membrane is selective in nature and allows only the passage of cations. In this way, the OH^–^ ions cannot cross the membrane and the cathode region has high pH. But, metal cations cross this membrane through migration under the action of the electric field and get precipitated after reaction with OH^−^ ions near the cathode region [28]. These two methods have achieved good results in soil restoration. In this study, pH of MSWI fly ash is pretreated by soaking in nitric acid solution to enhance the removal efficiency of HMs by electrokinetic remediation. The present experiment is aimed at characterization of MSWI fly ash, effect of nitric acid pretreatment and time periods on removal efficiency. Moreover, the removal mechanism of HMs is also discussed.

## 2. Material and methods

### 2.1. Materials

The MSWI fly ash samples were brought from TongXing Waste Power Generation Plant situated in TongXing town, Beibei district, Chongqing, China. The samples were dried at 105°C for 12 h.

### 2.2. Physical and chemical analysis

Original MSWI fly ash samples were subjected to particle size distribution analysis which was performed by a laser particle size analyser (Microtrac, S3500). In order to improve the homogeneity among particles, the samples were ground for 4 h by a ball grinding mill and sieved through a 200-mesh (75 µm). Semi-quantitative elemental analysis of the fly ash samples was performed by X-ray fluorescence (XRF, 1800CCDE). Total amount of heavy metals was determined by a flame atomic absorption spectrophotometer after microwave digestion of fly ash samples. The leaching toxicity acetic acid buffer solution method (HJ/T300-2007) was used to analyse the leaching toxicity.

### 2.3. Experimental device

**Figure 1.**
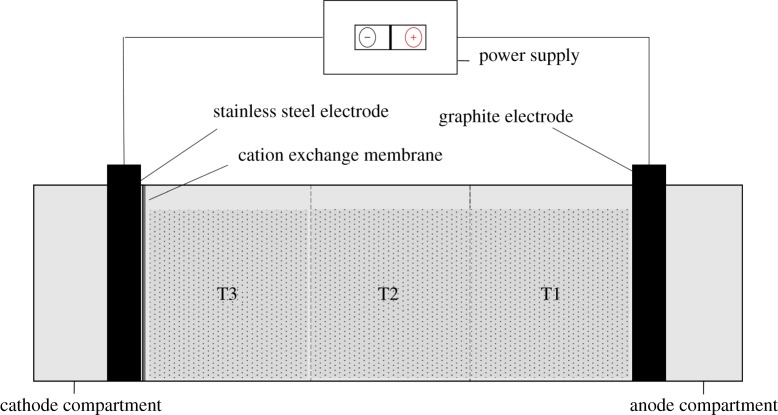
Electrokinetic remediation experimental set-up.

The electrokinetic remediation experiment was set up in a rectangular glass reactor which has three compartments, i.e. a sample region and two electrode regions; however, the sample region with dimensions of 10 × 7 × 8 cm is divided into three sections (T1, T2 and T3) towards anode to cathode for all experiments (figure 1).

### 2.4. Experimental layout

In the current study, two experiments were conducted to evaluate the removal efficiency of HMs from MSWI fly ash. In the first experiment, the MSWI fly ash was pretreated (soaked) with different nitric acid concentrations (A1, A2, A3 and A4) for 36 h to reduce its alkalinity. Afterwards, nitric acid pre-treated MSWI fly ash samples were subjected to electrokinetic remediation for 10 days (proposing/reaction time) to analyse the effect of different concentrations of nitric acid on the removal efficiency of HMs. In the second experiment, different proposed/reaction time periods were used to evaluate the electrokinetic remediation of HMs from fly ash pretreated with nitric acid. The complete layouts of experiments 1 and 2 are given in tables 1 and 2, respectively.

**Table 1.** The layout of the nitric acid experiment. (The liquid–solid is ratio of deionized water (V) to fly ash (m) and concentration is the ratio of nitric acid to the total solution on volume basis.)

	pretreatment experiment			electrokinetic remediation	
no.A-	liquid–solid ratio (m/v)	concentration (v/v) (%)	soaking time (h)	proposing time (days)	voltage gradient (V cm^−1^)
A1	3 : 5	0	36	10	1.5
A2	3 : 5	10	36	10	1.5
A3	3 : 5	20	36	10	1.5
A4	3 : 5	30	36	10	1.5

**Table 2.** The experimental layout of proposing time. (The liquid–solid is the ratio of volume of deionized water to mass of fly ash, and concentration is the ratio of volume of nitric acid to total volume of solution.)

	pretreatment experiment			electrokinetic remediation	
no. S-	liquid–solid ratio (m/v)	concentration (v/v) (%)	soaking time (h)	proposing time (days)	voltage gradient (V cm^−1^)
S1	3 : 5	30	36	4	1.5
S2	3 : 5	30	36	8	1.5
S3	3 : 5	30	36	12	1.5
S4	3 : 5	30	36	16	1.5

### 2.5. Leaching experiment and removal efficiency

Total amount of heavy metals was determined by a flame atomic absorption spectrophotometer after microwave digestion of fly ash samples. Leaching toxicity acetic acid buffer solution method (HJ/T300-2007) was used to analyse the leaching toxicity in both pre- and post-experiment samples. The leaching toxicity of HMs (Cu, Zn, Pb and Cd) was evaluated according to table 3 defined by the secondary standards for environmental soils in residential land (GB15618-2008). The leaching toxicity ratios of T1, T2 and T3 sample regions were calculated according to C/C_0_; C and C_0_ are leaching toxicities of pre- and post-experiment samples, respectively. The removal rates in each sample region were determined by the following formula:

$$y=\frac{U_{0}-U}{U_{0}}\times 100\text{\% },$$

where *y* represents removal rate, *U*_0_ is the total content of HMs in the original MSWI fly ash sample and *U* represents the total content of HMs after electrokinetic remediation.

**Table 3.** Total content (mg kg^−1^) and leaching toxicity (mg l^−1^) of HMs in MSWI fly ash.

item	Cu	Zn	Pb	Cd
the total content	757	6342	1687	153
GB15618-2008 II	300	500	300	10
the total content	2.5	12.684	5.623	15.3
GB15618-2008 II				
the leaching toxicity	2.796	22.400	3.935	1.893
GB16889-2008	40	100	0.25	0.15
the leaching toxicity	0.0699	0.2240	15.740	12.62
GB16889-2008				

## 3. Results and discussion

### 3.1. The properties of fly ash

**Figure 2.**
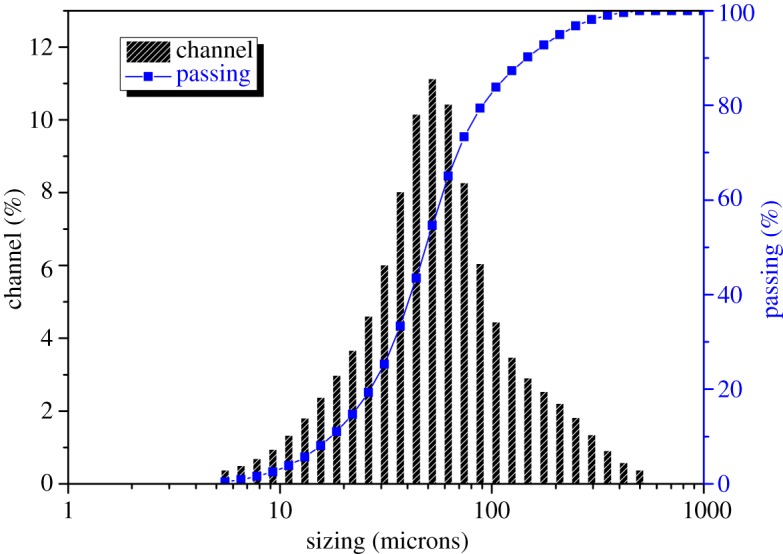
Particle size distribution of MSWI fly ash.

The particle size distributions of MSWI fly ash samples (figure 2) show that particles of fly ash are in the range of 5–600 µm. Furthermore, D10, D90 and D50 have particle sizes of 17.51, 146.1 and 48.71 µm, respectively. The semi-quantitative elemental results of the MSWI fly ash samples are shown in table 4. It can be seen that the major elements in MSWI fly ash are Ca, O, Cl, Na, K and Si. Zn, Pb, Cd and Cu are the main HM ions present in fly ash with a cumulative percentage of 0.9773%. The total amount and leaching toxicity of HMs from MSWI fly ash are shown in table 3. It can be seen that these metal ions have greatly exceeded the secondary standards used for environmental soils in residential land (GB15618-2008). The leaching toxicity of Pb and Cd in the fly ash has also exceeded the pollution control standards used for landfill sites.

**Table 4.** Semi-quantitative elemental analysis (%) of MSWI fly ash.

Ca	O	Cl	Na	Si
35.5754	33.9525	11.9567	4.2485	3.2786
K	S	Mg	Fe	Al
2.8724	2.3344	1.5514	1.1378	1.0806
Zn	P	Ti	Pb	Cd
0.7312	0.4438	0.2846	0.1628	0.1107
Cu	Br	Sr	Mn	Ni
0.0833	0.0749	0.0570	0.0554	0.0078

### 3.2. Macro phenomenon

#### 3.2.1. Macro phenomena of pretreatment

No reaction was observed upon mixing of water and MSWI fly ash samples, but bubbling phenomenon is observed after mixing the nitric acid and MSWI fly ash. The intensity of bubbling depends upon the concentration of acid. This might occur because MSWI fly ash has a large number of carbonate ions which react with hydrogen ions and release CO_2_. Similar phenomenon was observed by [29].

#### 3.2.2. Macro phenomena of electrokinetic remediation

In both experiments, some similar phenomenon is observed during electrokinetic remediation which is (i) bubbling, (ii) precipitation and (iii) disruption of exchange membrane. (i) Bubbling is observed in the cathode and anode regions after supplying the voltage which causes the electrolysis of water and release of Cl gas. The cathodic and anodic reactions are given below:

$$\begin{array}{ll} \text{cathodic reaction}: & 2{\text{H}}_{2}\text{O}+2{\text{e}}^{-}\to 2\text{O}{{\text{H}}^{-}}^{}+{\text{H}}_{2}↑\\ \text{anodic reaction:} & {\text{H}}_{2}\text{O}-2{\text{e}}^{-}\to 2{\text{H}}^{+}+\frac{1}{2}{\text{O}}_{2}↑\\ & 2{\text{Cl}}^{-}-2{\text{e}}^{-}\to {\text{Cl}}_{2}↑, \end{array}$$

(ii) A large amount of precipitates appeared on the stainless-steel cathode along with its compartment. The cathode generates OH^−^ ions which react with metal ions migrated from the sample area and cause precipitation. (iii) High current results in water splitting and polarization of ions which leads to the rupture of the exchange membrane at the cathode and causes escape of OH^−^ [30].

In addition to these phenomena, the hardening and compactness of MSWI fly ash is observed in sample regions. Hardness depends upon the concentration of acid, the more acid the less the compactness. So, it is obvious that degree of hardness is pH dependent. This hardness is developed because of the disruption of the cation exchange membrane which has released more OH^−^ ions towards the sample area and causes the precipitation of HMs.

### 3.3. Nitric acid pretreatment experiment

#### 3.3.1. Change in current over time

**Figure 3.**
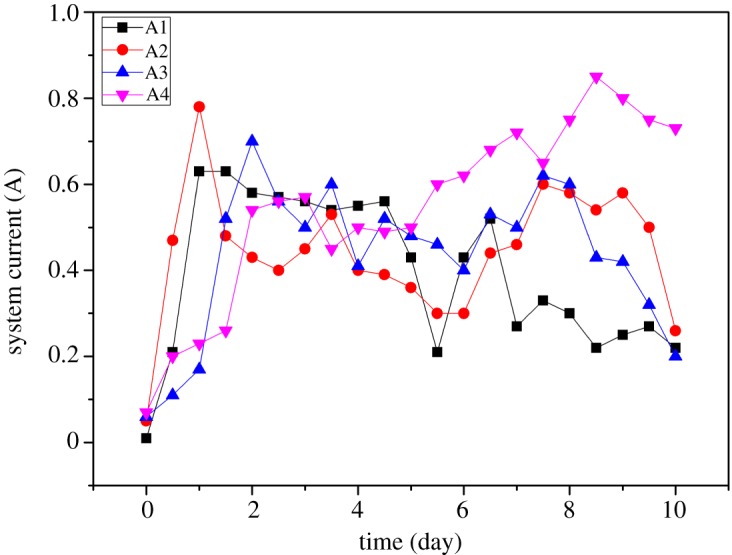
Change in current versus time (nitric acid experiment).

The variation in electrical current under different pretreatment conditions is shown in figure 3. The current in a system is defined as the quantity of ions moving through the sample region in a unit time. Generally, the current is increased at initial stages and then decreased with the passage of time in all groups (A1, A2, A3 and A4). The increase in current with the passage of time is not significant at initial stages because of the high buffering capacity of MSWI fly ash. But, regarding the decreasing trend of current, the main reason is the rupture of the cation exchange membrane which releases more OH^−^ ions. As a result, the precipitation/immobilization of free HM ions occurs which increases the current resistance. In the beginning, the current in group A1 is slightly higher than that in other three groups which are pretreated with nitric acid. It is because of more release of free ions in the presence of acid. However, the current is observed in the following order A1 < A2 < A3 < A4 on the 10th day. It is because of the release of H^+^ ions in the presence of an electric field which moves towards the sample area and releases more ions. Hence, A4 with a maximum concentration of acid has more current on the 10th day because of a higher concentration of free ions as compared to other groups.

#### 3.3.2. Change in pH with respect to time

**Figure 4.**
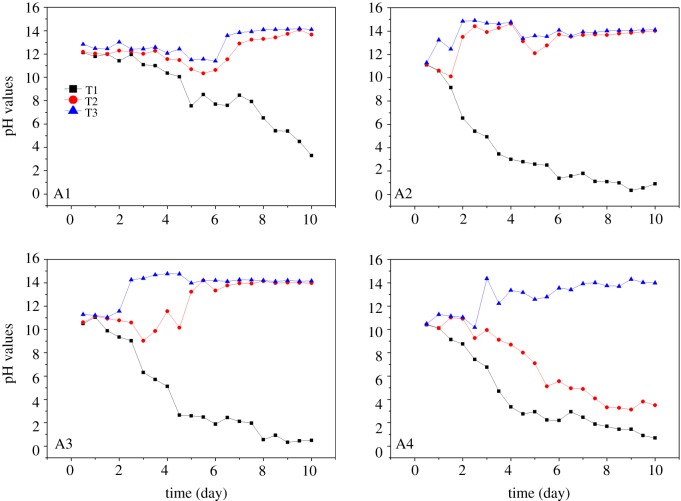
Change in pH versus time (nitric acid experiment).

The adsorption, desorption, precipitation and dissolution of metal ions are highly dependent on pH. In this way, the pH affects the electro-osmotic speed and the polarization process during electrokinetic remediation. Therefore, pH analysis over time is a necessary parameter. The pH values after pretreatment are 12.13, 11.25, 10.58 and 10.23 in groups A1, A2, A3 and A4, respectively. There is no significant difference in pH among the groups. This is because of the high buffering capacity of MSWI fly ash and it is difficult to decrease the pH values. The pH trend of the electrokinetic remediation experiment is shown in figure 4. Similarly, the gradual decrease and increase in pH is observed near the anode (T1) and cathode (T3) regions with the passage of time (in all groups). It is owing to the generation of OH^−^ ions at the cathode and H^+^ ions at the anode. The nitric acid soaking effect can be judged in the T2 sample region. As can be seen (figure 4), in group A4, T2 has more similarities with T1 sample region. The possible reason of this trend in the T2 region is the different concentration of acid which consumes carbonates and hydroxides to various levels in MSWI fly ash. Resultantly, pH gradient is changed owing to the reduced buffering capacity of MSWI fly ash.

#### 3.3.3. Leaching efficiency

The leaching efficiency of Cu, Zn, Pb and Cd in three sample regions (T1, T2 and T3) is shown in table 5. It can be seen that the sample region with lower pH values has higher leaching toxicity of HMs because higher concentration of H^+^ ions causes more release of HMs by exchanging with them. Therefore, the average leaching toxicity ratio of HMs is higher in A4 group when compared to other three groups. Similarly, the T1 region near the anode has higher desorption/release of HMs. After 10 days, the rate of desorption is greater than migration rate of HM ions which results in higher accumulation of free HM ions and also higher leaching toxicity ratios.

**Table 5.** Effect of nitric acid on leaching toxicity ratio.

	the ratio of leaching toxicity (C/C_0_)											
	Cu			Zn			Pb			Cd		
concentration	T1	T2	T3	T1	T2	T3	T1	T2	T3	T1	T2	T3
0% (A1)	0.600	0.424	0.242	0.904	0.683	0.268	0.527	0.520	0.382	0.801	0.506	0.240
10% (A2)	3.669	0.181	0.174	1.165	0.045	0.073	3.808	0.420	0.374	0.894	0.090	0.109
20% (A3)	3.246	0.162	0.158	1.149	0.018	0.014	1.944	0.399	0.418	0.771	0.057	0.053
30% (A4)	5.678	0.702	0.163	1.231	0.981	0.018	3.307	0.633	0.427	0.949	0.852	0.055

In landfill, acidic leachate is produced which can reduce the buffering ability of fly ash and ultimately increase the leaching toxicity. Similarly, higher leaching toxicity is observed in fly ash samples treated with a higher percentage of acid (A4 = 30%). Therefore, the A4 group is used in the second experiment to check the effect of time on the removal efficiency of HMs during electrokinetic remediation.

### 3.4. Experiments with different proposing times

In this section, the A4 group is subjected to electrokinetic remediation and the effect of reaction time on different parameters, i.e. current, pH and leaching efficiency is analysed.

#### 3.4.1. Change in current over time

**Figure 5.**
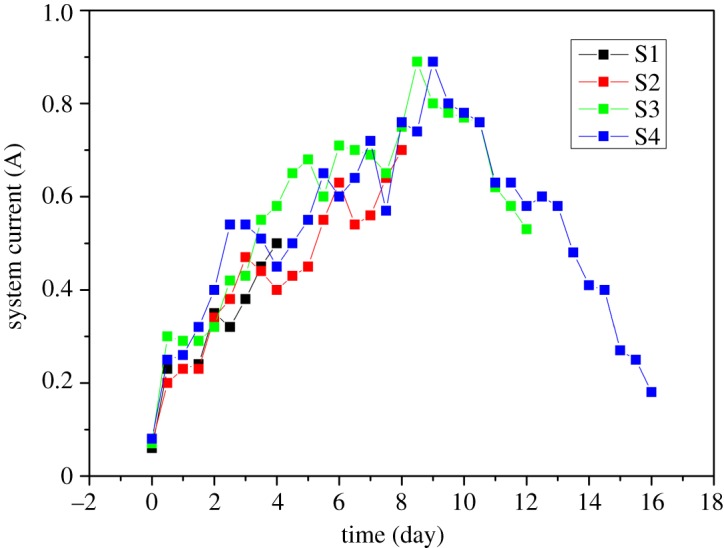
Change in current versus time (proposed time experiment).

Changes in current with respect to time are evaluated which are shown in figure 5. Generally, the current is increased first and then decreased in all groups (S1, S2, S3 and S4), but the highest current intensity is observed on the ninth day. The reasons regarding current changes have already been discussed in §3.3.1.

#### 3.4.2. Change in pH with respect to time

**Figure 6.**
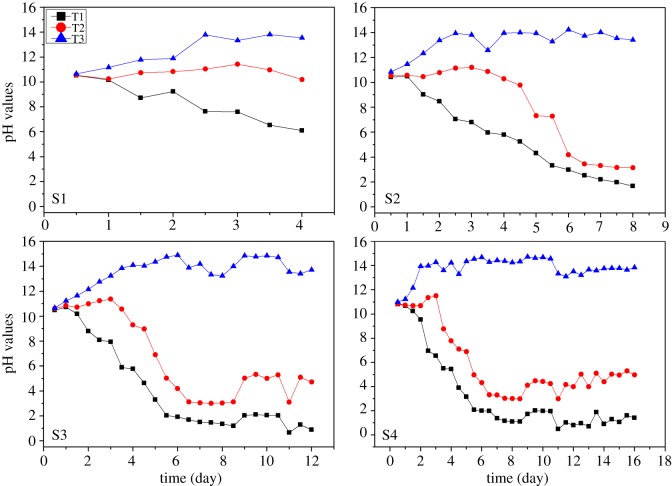
Change in pH versus time (proposed time experiment).

Influence of time on pH during electrokinetic remediation is shown in figure 6. Initially, the decrease or increase in pH is observed which depends upon the respective electrodes owing to the generation of H^+^ and OH^−^ ions. The variations in pH are observed up to a certain period of time, then the pH of each region is stabilized with the advancement of time. Basically, H^+^ and OH^−^ ions are migrated towards the sample region under the action of an electric field and causing the change in pH gradient. The reaction slows down with respect to time which results in pH stabilization.

#### 3.4.3. The leaching efficiency

Leaching efficiency is greatly influenced by reaction/proposing time which can be seen in table 6. In the S2 (8 d) group, a higher leaching toxicity ratio of Cu and Pb is observed in the T1 region. The Zn and Cd leaching toxicity ratio is maximum on the fourth day (S1) in the T1 region. It shows that different HMs require different pH to desorb/release which varies with time. The minimum leaching toxicity ratios of Cu (0.174), Zn (0.269), Pb (0.404) and Cd (0.083) are observed after 12 days. It is concluded that the extended proposed time has increased the removal efficiency of HMs. Additionally, electrokinetic remediation of HMs requires three processes, i.e. acidification, desorption and migration to enhance removal efficiency.

**Table 6.** Effect of proposing time on leaching toxicity.

	the ratio of leaching toxicity (C/C_0_)											
	Cu			Zn			Pb			Cd		
proposing time	T1	T2	T3	T1	T2	T3	T1	T2	T3	T1	T2	T3
4 d (S1)	1.752	1.740	0.185	1.158	1.210	0.230	1.049	1.050	0.427	1.091	1.054	0.132
8 d (S2)	4.464	0.470	0.088	1.255	0.987	0.026	2.533	0.597	0.398	1.056	0.654	0.035
12 d (S3)	0.366	0.084	0.071	0.765	0.021	0.021	0.393	0.418	0.401	0.164	0.040	0.044
16 d (S4)	0.276	0.540	0.100	0.727	1.039	0.039	0.411	0.625	0.425	0.136	0.758	0.067

### 3.5. Removal mechanisms of heavy metals

#### 3.5.1. Removal rate of total content

**Figure 7.**
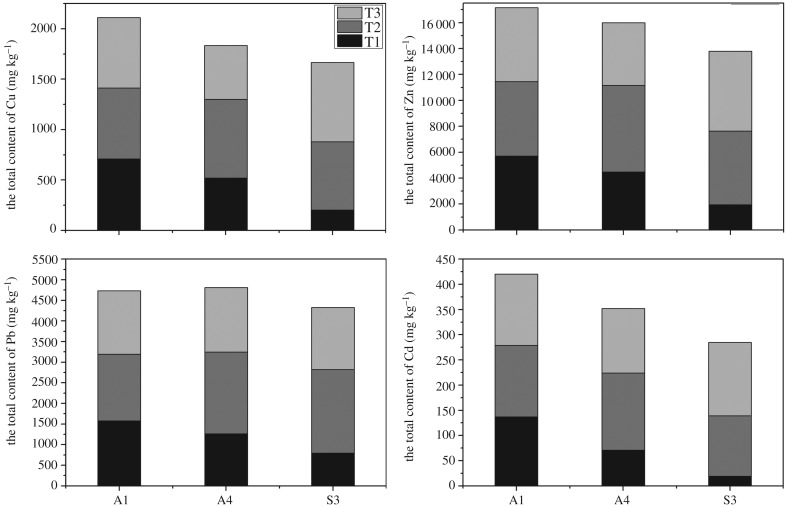
Total content of HMs in different regions.

The removal rate of total content of HMs is compared among group A1(10 days with no acid), group A4 (10 days with 30% acid) and group S3 (12 days with 30% nitric acid). The average removal rate of Cu, Zn, Pb and Cd is higher in group S3 followed by A4 and A1 which can also be seen in table 7. So, it is concluded that fly ash pretreated with 30% nitric acid subjected to longer period of time (12 d) can enhance the electrokinetic remediation process. Additionally, total content of Cu, Zn, Pb and Cd in three sample regions (T1, T2 and T3) within each group is also analysed and presented in figure 7. A higher concentration of HMs is observed in the T3 region which is near to the cathode because of a higher release and migration of HM ions from anode to cathode owing to the electric field.

**Table 7.** Average removal rate of total content.

	average removal rate			
concentration × proposing time	Cu (%)	Zn (%)	Pb (%)	Cd (%)
0% × 10 (A1)	7.13	9.87	6.56	8.50
30% × 10 (A4)	19.20	15.92	4.94	23.32
30% × 12 (S3)	26.64	27.53	14.44	37.91

#### 3.5.2. Phase changes

**Figure 8.**
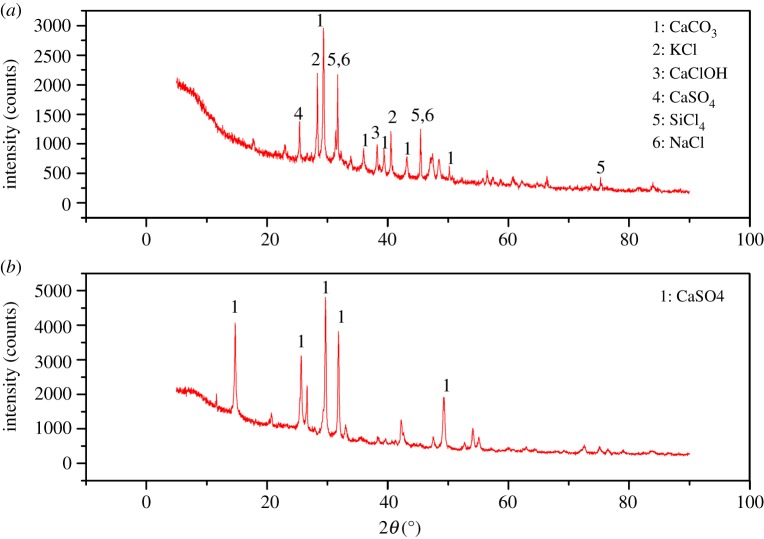
The XRD analysis of MSWI fly ash samples before remediation (*a*) and after remediation (*b*).

The mineralogical/phase analysis of raw MSWI fly ash and the S3-T1 sample is carried out by X-ray diffraction (XRD) (Shimadzu XRD-6000). It can be seen (figure 8*a*) that raw MSWI fly ash has CaCO_3_ and CaClOH phases which are the main reasons of the high buffering capacity of MSWI fly ash. Most of these species are dissolved after electrokinetic remediation which can be seen in figure 8*b*. Therefore, migration of metal ions is more efficient in the sample region.

#### 3.5.3. Changes in the morphology of heavy metals

**Figure 9.**
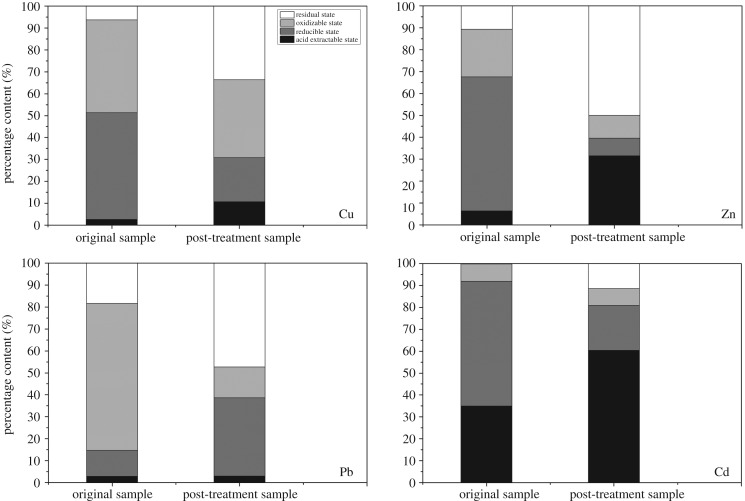
The BCR morphological analysis of HMs.

MSWI fly ash is a heterogeneous material and, therefore, HMs exist as complex, precipitated and dissolved forms. Hence, the original MSWI fly ash and S3-T1 samples are subjected to morphological analysis via a modified extraction method developed by the European Community Bureau of Reference (BCR). Generally, the removal mechanisms depend upon different morphologies of HMs in electrokinetic remediation. The HMs are categorized into four states: (i) an acid extractable state released in an acidic environment, (ii) a reducible state form complex with iron and manganese oxide which is released in reductive conditions, (iii) oxidizable states are bounded with organic matter and sulfide compound which can be released in oxidizing environment, and (iv) a residual state existing as a mineral bounded form and released upon weathering. The former three states can easily release in their respective conditions when compared to the fourth state which is highly immobile. As shown in figure 9, the residual fraction of HMs is significantly increased after the experiment. This is owing to the transformation and reduction of HMs in other morphologies that have higher environmental risk; but, the decrease in reducible and oxidizable fractions indicates that the removal efficiency of HMs in these states is enhanced by electrokinetic remediation with acid pretreatment. The increase in acid extractible extraction fraction is owing to the release and transformation of reducible and oxidizable states into acidic states. Acidic pretreatment has a strong effect on these two states, which are easily released during electrokinetic remediation. Moreover, the considerable fraction of HMs in an acid extractable state after the experiment indicate that although a great part of the total content of HMs was removed, there is also the potential to improve the removal rates by further extending the reaction time or by combining this with other effective techniques, which we can study in the future. The results indicate that significant amounts of total content of HMs are removed along with acidic fraction. Moreover, the removal rate can also be increased by extending reaction time or by combining this work with other techniques.

## 4. Conclusion

The main purpose of this experiment is to increase the desorption of HM ions from MSWI fly ash which can easily migrate from anode to cathode and get precipitated. In this way, the removal efficiency of HMs can be increased through electrokinetic remediation. The influence of pH on the desorption of HMs can be judged by a leaching experiment and electric current intensity. A higher leaching toxicity ratio is observed in the A4 group (30% nitric acid) when compared to the A1 group which is not treated with nitric acid after 10 days. Similarly, a higher electric current is observed in group A4 owing to the presence of a higher number of free ions on the 10th day. Therefore, the A4 group is subjected to the second experiment in which the effect of time on removal efficiency of HMs through electrokinetic remediation is analysed. A higher removal rate or lower leaching toxicity ratio is observed in the S3 group (12 d). Moreover, total content of HMs are reduced and residual states (highly immobilized) of HMs are increased which has lowered the environmental risk. It is owing to the change in morphologies of HMs, i.e. from an oxidizable and reducible state to a more easily removable state. Keeping these points in view, it is concluded that the acidification process has increased the desorption of ions from MSWI fly ash. In this way, the electrokinetic remediation mechanism is enhanced because higher desorption of ions facilitates its migration and precipitation of metal ions at the cathode region. Consequently, lower leaching toxicity ratios are observed in group S3 (12 d) owing to higher precipitation of metal ions after reacting with OH^−^ ions.

## Data accessibility

All data and analysis scripts associated with this analysis can be downloaded from Dryad (http://dx.doi.org/10.5061/dryad.q91436s) [31].
